# Aqueous extract from *Mangifera indica* Linn. (Anacardiaceae) leaves exerts long-term hypoglycemic effect, increases insulin sensitivity and plasma insulin levels on diabetic Wistar rats

**DOI:** 10.1371/journal.pone.0227105

**Published:** 2020-01-08

**Authors:** Gustavo Roberto Villas Boas, João Marcos Rodrigues Lemos, Matheus William de Oliveira, Rafael Claudino dos Santos, Ana Paula Stefanello da Silveira, Flávia Barbieri Bacha, Caren Naomi Aguero Ito, Ediane Bortolotte Cornelius, Fernanda Brioli Lima, Andrea Marisa Sachilarid Rodrigues, Nathália Belmal Costa, Felipe Francisco Bittencourt, Fernando Freitas de Lima, Marina Meirelles Paes, Priscila Gubert, Silvia Aparecida Oesterreich

**Affiliations:** 1 Research Group on Development of Pharmaceutical Products (P&DProFar), Center for Biological and Health Sciences, Federal University of Western Bahia, Barreiras, Bahia, Brazil; 2 Faculty of Health Sciences, Federal University of Grande Dourados, Dourados, Mato Grosso do Sul, Brazil; 3 Faculty of Health Sciences, University Center of Grande Dourados, Dourados, Mato Grosso do Sul, Brazil; 4 Department of Biochemistry, Laboratory of Imunopathology Keizo Asami, Federal University of Pernambuco, Recife, Brazil; The University of Manchester, UNITED KINGDOM

## Abstract

**Background:**

Diabetes mellitus is one of the most common todays public health problems. According to a survey by the World Health Organization, this metabolic disorder has reached global epidemic proportions, with a worldwide prevalence of 8.5% in the adult population.

**Objectives:**

The present study aimed to investigate the hypoglycemic effect of aqueous extract of *Mangifera indica* (EAMI) leaves in streptozotocin-induced diabetic rats.

**Methods:**

Sixty male rats were divided into 2 groups: Normoglycemic and Diabetic. Each group was subdivided into negative control, glibenclamide 3 or 10 mg/kg, EAMI 125, 250, 500, and 1000 mg/kg. Intraperitoneal injection of streptozotocin 100 mg/kg was used to DM induction. The hypoglycemic response was assessed acutely after two and four weeks of treatment. After a 6-hour fasting period, the fasting blood glucose of animals was verified, and 2.5 g/kg glucose solution was orally administered. The insulin tolerance test and plasma insulin levels assessment were performed in the morning after fasting of 12 to 14 hours.

**Results and conclusion:**

The chemical analysis of EAMI showed high levels of phenolic compounds. There was no significant difference in fasting blood glucose between normoglycemic and diabetic groups, and that EAMI did not have an acute effect on diabetes. After two and four weeks of treatment, the extract significantly reduced blood glucose levels, exceeding glibenclamide effects. EAMI was effective in maintaining the long-term hypoglycemic effect, as well as, significantly increased the sensitivity of diabetic animals to insulin and the plasma insulin level.

## 1. Introduction

Diabetes mellitus (DM) has reached a pandemic condition, affecting 8.5% of adults worldwide. Regarding global epidemiology, in 2017, the prevalence of DM in North America and the Caribbean was 11%, the highest worldwide incidence of the disease. An intermediate incidence was observed in Southeast Asia, with 10.1% of patients diagnosed with DM. The African region presents the lowest prevalence of 4.4 which was attributed to low levels of urbanization and obesity incidence of obesity and higher rates of infectious diseases [1,2].

DM is a metabolic disease characterized by hyperglycemia that results from defects in insulin action and/or secretion. Also, disorders involving the carbohydrates, fats, and proteins' metabolism are associated with dysfunctions in several organic systems [3–5]. Three schemes are currently used to classify the disease: (1) based on pathophysiology, (2) based on a specific genetic defect or (3) based on another typical phenotype [6]. Thus, DM can be mainly divided into type 1 DM (5–10% of cases) or type 2 DM (approximately 90% of cases) [7]. Harmful effects of DM can be controlled and suppressed when identified in the early stages of the disease. Therefore, proper classification is an essential task in diabetes prediction and diagnosis [8].

Different types of synthetic drugs, such as biguanides, sulfonylureas, thiazolidinediones, among others, are currently available therapies to DM [4]. Long-term treatments lead to a longer and healthier life, alleviating symptoms, and reducing the risks inherent to DM [9].

Although promising DM control, antidiabetic drugs have adverse effects described in the literature. Metformin, a biguanide, can cause abdominal discomfort, anorexia, diarrhea, nausea, renal hypoperfusion, and lactic acidosis with severe renal impairment. Thiazolidinediones cause anemia, insomnia, headache, dizziness, gastrointestinal disorders, hematuria, proteinuria, hypoglycemia, among others [10,11]. Sulphonylureas can cause mild headaches, increased food intake, gastrointestinal disturbances, weight gain, severe cardiovascular disorders, and other fatal complications [12–16]. The most severe adverse effects related to medications currently used in DM pharmacotherapy are rare. However, all drug used must be guided by a qualified professional.

Studies on medicinal plants with hypoglycemic effects are crucial in the prospection of new antidiabetic therapies with lower adverse effects. About 25% of the drugs prescribed in the world derive from plants already used in traditional medicine [17]. Such plants, popularly used for years, are the preferred source of botanical material for the investigation of new effective drugs in therapeutics [18].

Scientific interest in the biological properties of *Mangifera indica* L. (Anacardiaceae) as a hypoglycemic agent has increased in recent years. Its leaves, and ashes of leaves, are widely used in folk medicine for DM treatment, inflammation, diarrhea, dysentery, leukocytosis, and burns [19,20,21–28,29]. Ethnopharmacological studies have demonstrated that *M*. *indica* has been used for a long time in the worldwide treatment of DM [19].

The Anacardiaceae family consists of about 76 genera and 600 species. Phytochemical studies of the different Anacardiaceae species revealed the presence of flavonoids, terpenes, steroids, phenolic lipids, and xanthones [30]. The *M*. *indica* species is popularly known as mango, native to tropical Asia and successful cultivated in sub-tropical conditions [31,32]. It is a large fruit tree, perennial, and anchored by a long main root. Leaves are usually green, flowers are red or yellow-green, and fruits vary in size, shape, and color. Among *M*. *indica* parts, leaves are one of the most important sources of phenolic compounds and mangiferin, surpassing the bark [33].

Although research has shown that the aqueous extract from *M*. *indica* leaves exhibits hypoglycemic activity in animal models [25,26,29,34,35], there is currently no experimental evidence available in the literature regarding the maintenance of the acute hypoglycemic effect, which characterizes antidiabetic therapy. Therefore, the present study aimed to investigate the possible maintenance of the hypoglycemic effect of the aqueous extract from *M*. *indica* leaves on streptozotocin-induced diabetic rats.

## 2. Methods

### 2.1. Botanical material and extract preparation

*M*. *indica* leaves (2500 g) were collected on August 2016 in Dourados, Mato Grosso do Sul, Brazil (latitude 20° 26 '34 "S and longitude 54° 38' 47" W) according to authorization issued by the Brazilian Agency of the Environment (registration No. 61621–3—MMA/ICMBio/SISBIO) and an exsiccata (registration No. 3822) was deposited at the Plant Facility of the Federal University of Grande Dourados (UFGD), Mato Grosso do Sul, Dourados, MS, Brazil.

The botanical material underwent a process of natural drying and artificial drying in the oven with forced air circulation. Natural drying was performed according the to protocol proposed by Simões et al. (2010) [36]. Leaves were placed in the shade, in a clean, dry place, free of insects, and arranged on paper towel for moisture absorption for 7 days. Artificial drying was performed immediately after natural drying through Pardal PE 30 dehydrator with forced air circulation at approximately 40°C for 24 hours and then sprayed in GTM-2001 C forage crusher at the Laboratory of Bromatology—University Center of Grande Dourados (UNIGRAN).

Extraction was carried out by boiling 2000 g of pulverized vegetable material in 2 L of water for 4 hours, cooled, centrifuged, and the supernatant stored at 4°C. The extract was then lyophilized in FTS Systems TDS-00209-A lyophilizer and stored [37], resulting in 341.64 g of aqueous extract from *M*. *indica* leaves (EAMI). Based on the yield of the extract, the doses tested in the study (1000, 500, 250, and 125 mg/kg) are roughly equivalent to 4.5, 2.25, 1.125, and 0.5625 g of leaves, respectively.

### 2.2. Quantification of the total phenolic content

EAMI (100 μg) was dissolved in methanol solvent (1g/L), where 100 μL of *M*. *indica* solution was mixed with distilled water (1.0 mL) and 0.5 mL of folin-ciocalteu reagent (1:10 hgv/v). Finally, the sodium carbonate solution (2%, 1.50 mL) was added and manually shaken for 30 min, with intervals. Absorbance was measured at 765 nm using a spectrophotometer. Total phenolic concentration is expressed as gallic acid equivalent (GAE) in mg per gram of extract [38], using the calibration curve with gallic acid (y = -0.0315+51.94283x; R = 0.99438).

### 2.3. Tannin concentration

Tannin concentration was measured by the reaction of vanillin, according to Broadhurst and Jones (1978) [39], adapted by Agostini-Costa and colleagues (1999) [40], in which 5 ml vanillin, freshly prepared reagent was added to each test tube (in five times). Test tubes were preheated at 30°C for 30 min. Then, 1 ml of EAMI was added to each tube and vortexed for 30 sec. The reaction was maintained at 30° C for 20 minutes, followed by absorbance reading at 510 nm within a maximum period of 1 hour. Quantification was performed by means of external calibration curve, using catechin as standard (y = 0.16448 + 1.92361x; R = 0,99923). Results are expressed in mg of catechin equivalent (CE) per 1 g of sample.

### 2.4. Quantification of the flavonoid content

The protocol described by Lin and Tanf (2007) [38] was performed to quantify the flavonoid content. EAMI was solubilized in ethanol at 1000 μg mL -1. The following proportions were used for the trial: 1.5 mL of 95% ethyl alcohol, 2.8 mL of distilled water, 500 μL of sample, 100 μL of sodium acetate (NaC2H3O2.3H2O) 1 mol.L-1 and 100 μL of 10% aluminum chloride (AlCl3.6H2O). After 40 minutes of reaction at room temperature, readings were performed in a spectrophotometer at 415 nm.

For the blank, the same procedure was performed with the sample replacement by ethanol. The analytical curve (2.5, 5.0, 10.0, 20.0, 25.0, 50.0, 100.0, and 125.0 μg) was prepared using quercetin as standard. The experimental procedure performed with the standard was the same used for samples. Data were submitted to linear regression, obtaining the equation of the line, which had its data used in the calculation of real samples. The results were expressed in milligrams of quercetin per gram of extract, and tests were performed in triplicate.

### 2.5. Extract fractionation

For extract fractionation, the protocol described by Villas-Boas et al. (2018) [41] was adapted. The EAMI extract (50.66 g) was dissolved in water: ethanol (3: 1 v/v) and partitioned with organic solvents (acetone and ethyl acetate). The acetone fraction of EAMI was fractionated on a chromatographic column using silica gel (0.063–0.200 mm, Merck) in gradient solvent system (acetone and ethyl acetate) and the obtained fractions were analyzed by comparative thin-layer chromatography (TLC, Macherey-Nagel) and 8: 2 v/v hexane: acetone on elution. Fractions 57–75 were pooled and submitted to purification using preparative thin-layer chromatography (TLC) with 20x20 cm plates on silica gel. The elution was performed using 10:1 v/v hexane: acetone, yielding three samples (**F-1**, 2.6 mg, **F-2**, 2.2 mg, and **F-3** 2.8 mg). The ethyl acetate fraction of EAMI was fractionated on a chromatographic column using silica gel (0.063–0.200 mm, Merck) in gradient solvent system hexane: ethyl acetate (7: 3 v/v) (1), hexane: ethyl acetate (5: 5 v/v) (2), ethyl acetate (3) and ethanol (4), resulting in a fraction for each solvent system. Fractions were analyzed by TLC, denominated 1, 2, and 3 and submitted to TLC fractionation. Fractions 1 and 2 were eluted using 7: 3 v/v ethyl hexane: ethyl acetate, and fraction 3 was eluted with 4: 6 v/v ethyl hexane: ethyl acetate. From fraction 1, a solid of 1.9 mg (**F-4**) was obtained, and fraction 2, 1.7 mg (**F-5**) was obtained. Substances were identified using spectroscopic analyses (RMN ^1^H, ^13^C, and RMN 2D) and data obtained were compared to those described in the literature [42–46].

### 2.6. Drugs and treatments

Streptozotocin (STZ) (Sigma) 100mg/kg/day (i.p.) was used for diabetes induction, and 0.01 M citrate buffer pH 4.5 as vehicle. For the evaluation of the hypoglycemic activity, EAMI (125, 250, 500, and 1000 mg/kg) and glibenclamide (as positive control at dose of 3 mg/kg for normoglycemic animals and 10 mg/kg for diabetic animals) (Sanofi- Aventis Farmacêutica Ltda) were suspended in distilled water and orally administered (gavage). EAMI doses were determined based on safety standards identified from acute and short-term oral toxicity studies. The negative control group received saline.

### 2.7. Evaluation of the hypoglycemic activity, Insulin Tolerance Test (ITT) and insulinemia

#### 2.7.1. Animals and groups

Sixty male Wistar rats, 250–300 g, were obtained from the animal facility of the Federal University of Mato Grosso do Sul (UFMS). Animals were kept at the animal facility of the UNIGRAN Faculty of Health Sciences at 22 ± 2°C, 40–60% humidity, and 12-h light-dark cycle, with *ad libitum* access to water and food. All experimental procedures were developed in accordance with the ethical precepts of animal research and previously approved by the UNIGRAN Ethics Research Committee on Animal Use (protocol No. 004/2016).

Animals were divided into 2 groups: 1) normoglycemic group (n = 30) and 2) diabetic group (n = 30). Subsequently, each group was subdivided into (n = 5 / group): 1) negative control (NC); 2) Glibenclamide (GCM 3 mg/kg or 10 mg/kg), 3) EAMI 125 mg/kg; 4) EAMI 250 mg/kg; 5) EAMI 500 mg/kg and; 6) EAMI 1000mg/kg.

#### 2.7.2. Diabetes induction

Diabetes induction was performed according to the protocol proposed by Xu et al. (2006) [47]. Intraperitoneal injection of STZ 100mg/kg/day was given for three consecutive days. One week after induction, animals fasted for 6 hours for plasma glucose dosing, and animals with blood glucose levels less than 200 mg/dL were excluded from the diabetic group. In cases of absence of DM induction, other rats were submitted to the same treatment scheme to maintain the number of 5 animals per subgroup of the diabetic group.

#### 2.7.3. Hypoglycemic response evaluation in normoglycemic and diabetic rats

The hypoglycemic response was evaluated after six weeks of diabetes induction in 3 different moments: 1) acute response (immediately after the first administration of treatments); 2) after two weeks and; 3) after four weeks of treatment. For this, normoglycemic and diabetic animals remained fasted for 6 hours, and then fasting blood glucose levels were checked, and subsequently 2.5 g/kg body weight glucose solution diluted in drinking water was orally administered (gavage). Thirty minutes after receiving the glucose solution, normoglycemic animals underwent the following oral treatments: 1)NC, saline; 2) GCM 3mg/kg; 3) EAMI 125 mg/kg; 4) EAMI 250 mg/kg; 5) EAMI 500 mg/kg; 6) EAMI 1000 mg/kg. Diabetic animals received the same oral treatment and only for the positive control group, the glibenclamide dose was increased to 10 mg/kg body weight. Blood samples were collected from the caudal vein at 0, 2, 4, 6 and 8 hours after treatments. Plasma glucose levels were determined using the Accu-Chek® Active apparatus from Roche.

#### 2.7.4. Insulin Tolerance Test (ITT)

The test was performed in the morning after fasting from 12 to 14 hours after 4 weeks of treatment with EAMI. All animals in each group were anesthetized with sodium thiopental (40 mg/kg body weight) and, after confirming the anesthetic effect from the absence of caudal reflex to the pressure stimulus, 25 microlitres of blood was collected after tail section for measuring basal glycemia. The animals received an intraperitoneal injection of regular insulin solution diluted in saline (30 mU/100g body weight), and at 15, 30, 60, and 120 minutes after administration, 25 μl of blood were collected to glucose levels determining. Plasma glucose levels were assessed using Labtest® [48]. Subsequently, the percent glucose decay constant (K_ITT_%) was calculated according to the equation below:

$$K_{ITT\%}=\frac{0,693}{T_{1/2}}\times 100$$

At where: T_1/2_ = time required to reduce the basal glycaemia by half.

#### 2.7.5 Insulinemia

After four weeks of EAMI treatment, the animals were submitted to euthanasia through inhalation anesthesia with isoflurane followed by exsanguination. The blood collected was used for insulinemia analyses. Samples were centrifuged at 146 g for 3 minutes at 4° C, and the plasma was stored at -70° C. The procedure followed the basic principle of Radioimmunoassay (RIA), where there is competition between radioactive and nonradioactive antigen by a fixed number of antibody binding sites. The amount of 125I-insulin bound to the antibody is inversely proportional to the plasma insulin concentration. The labeled antigen-antibody complex formed was precipitated in polyethylene glycol 6000 (PEG 6000) and dosed in the gamma type counter (PerkinElmer®).

### 2.8. Statistical analyses

All results were expressed as mean ± standard error of the mean (SEM). One-way or two-way ANOVA, followed by Tukey's post hoc test, was used to evaluate fasting blood glucose and hypoglycemic response after treatments. Correlation between plasma glucose levels during ITT and KITT% was performed using the Spearman's correlation test. Data express mean ± SEM, and the significance level was defined as p <0.05. Statistical analyses were performed using Stats of Statistica software version 7.0 for Windows.

## 3. Results

**Fig 1:**
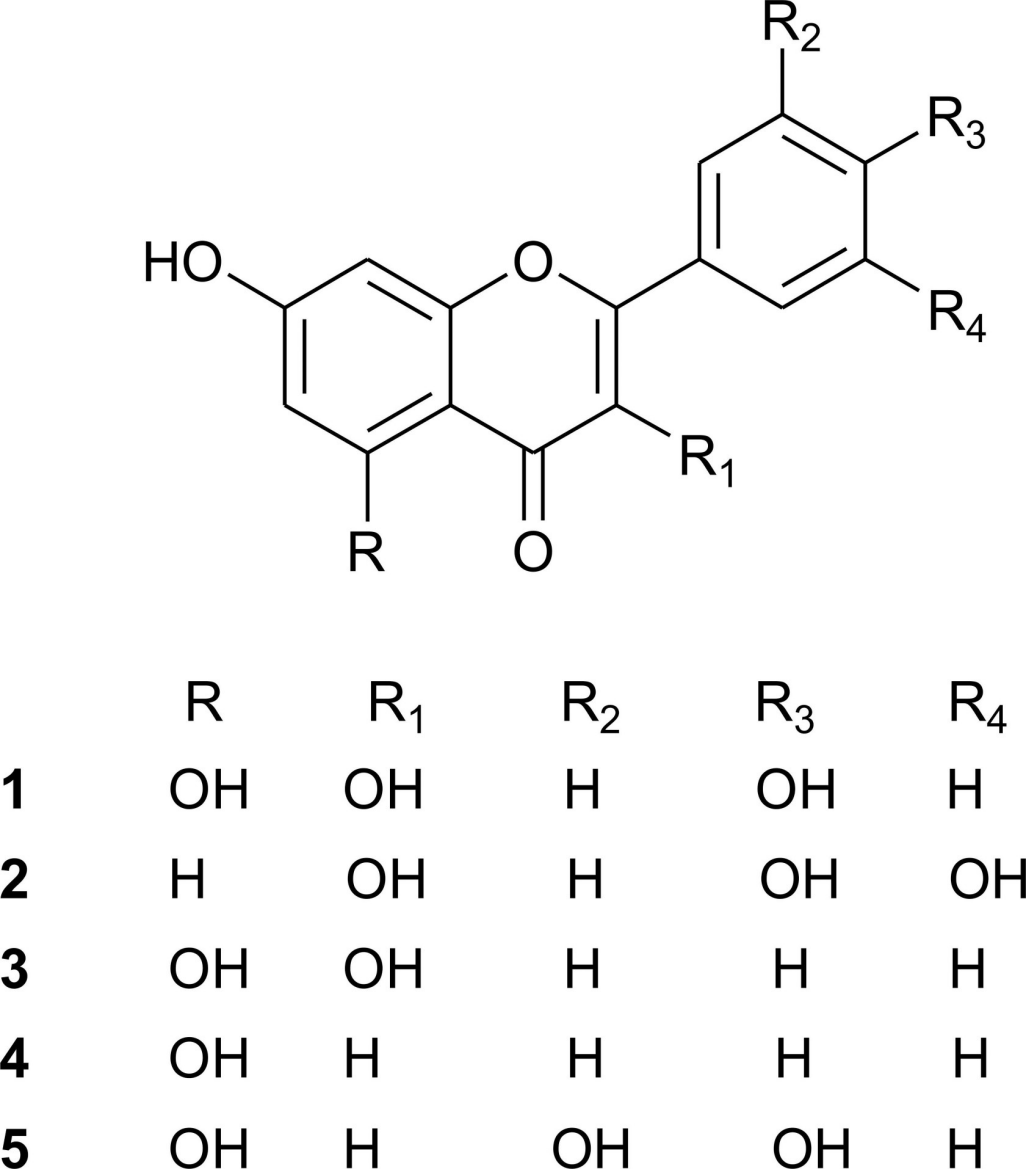
Structure of flavonoids isolated from *M*. *indica* leaves. **(1)** 5,7,4'-trihydroxyflavonol (kaempferol; **(2)** 7,3',4'-trihydroxyflavonol (fisetin); **(3)** 5,7-dihydroxyflavonol (galangin); **(4)** 5,7-dihydroxyflavone (Chrysin) and **(5)** 5,7,3’,4’-Tetrahydroxyflavone (Luteolin).

The chemical analysis of EAMI quantified high levels of total phenolic compounds (65.93 ± 0.40 mg GAE/g extract), total tannins (147.38 ± 2.74 mg CE/g extract) and flavonoid content of 237.44 mg of quercetin per gram of extract. Five flavonoids were isolated from the extract, three of which were obtained from the acetone fraction (**F-1** 5,7,4'-trihydroxyflavonol (Kaempferol); **F-2** 7,3',4'-trihydroxyflavonol (Fisetin) and **F-3** 5,7-dihydroxyflavonol (Galangin) and two from the ethyl acetate fraction (**F-4** 5,7-dihydroxyflavone (Chrysin) and **F-5** 5,7,3’,4’-Tetrahydroxyflavone (Luteolin) (Fig 1).

**Fig 2:**
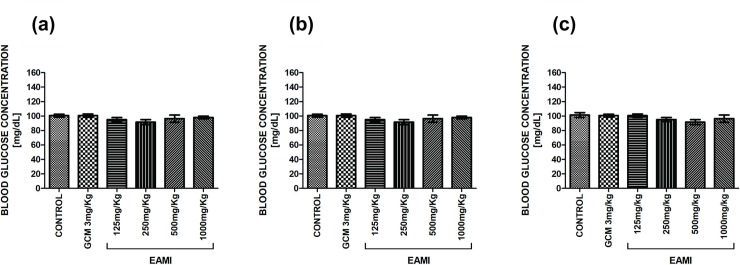
Fasting blood glucose of the normoglycemic group. (a) Acute test; (b) Test after two weeks of treatment; (c) Test after four weeks of treatment. Bars represent the mean ± SEM of the blood glucose level (one-way ANOVA, p <0.05, *post hoc* Tukey).

**Fig 3:**
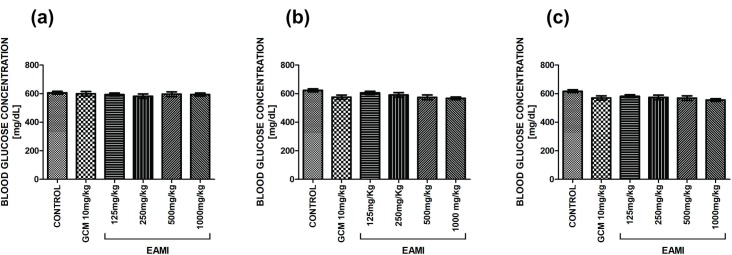
Fasting blood glucose of the diabetic group. (a) Acute test; (b) Test after two weeks of treatment; (c) Test after four weeks of treatment. Bars represent the mean ± SEM of the blood glucose level (one-way ANOVA, p <0.05, *post hoc* Tukey).

Statistical analysis showed that there was no significant difference in fasting blood glucose for both normoglycemic group (Fig 2A, 2B and 2C) and diabetic group (Fig 3A, 3B and 3C) in the acute tests and after two and four weeks of treatment, demonstrating that the animals had similar blood glucose levels before the start of treatment.

**Fig 4:**
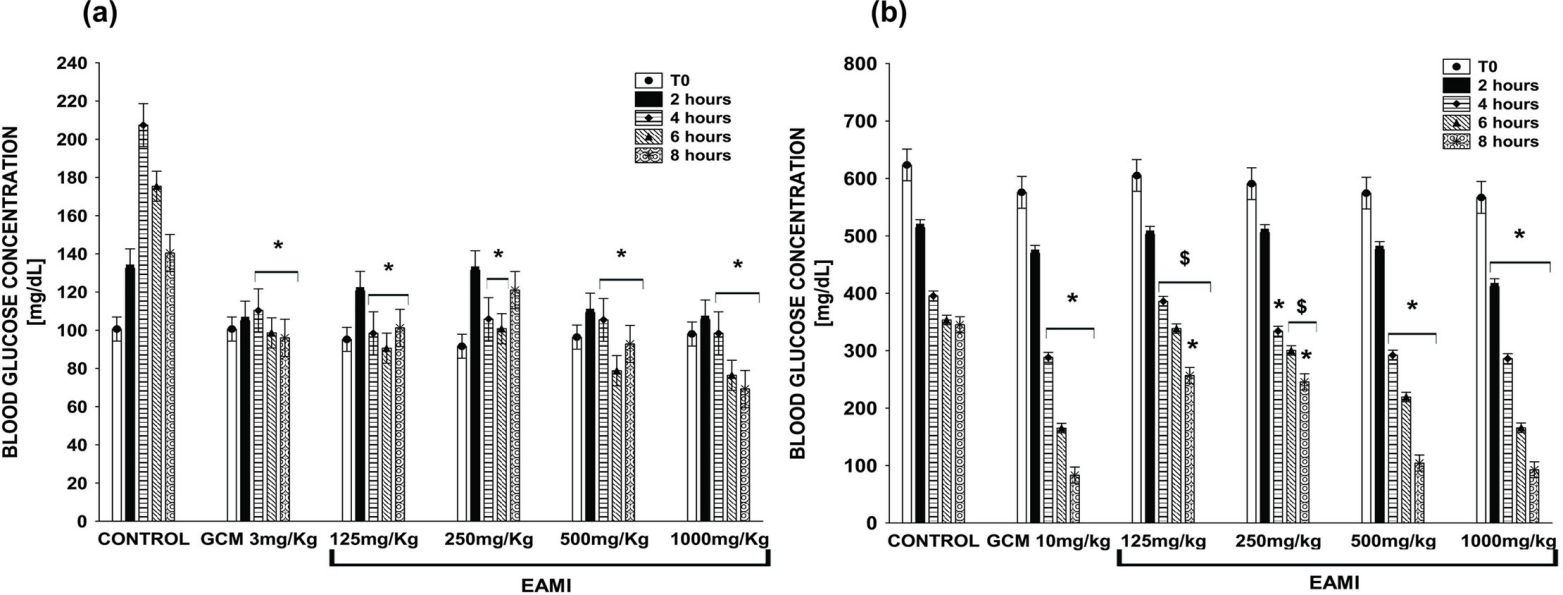
Plasma glucose level after two weeks of treatment. (a) Normoglycemic group; (b) Diabetic group. Bars express the mean ± SEM of the plasma glucose level. Symbols represent significant differences: * differs from the control group; $ differs from the GCM 10 mg/kg group (ANOVA for multivariate measures, p <0.05, *post hoc* Tukey).

The test performed to determine the acute hypoglycaemic effect of EAMI did not demonstrate significant changes in plasma glucose levels in normoglycemic and diabetic animals when compared to the control group and/or GCM 3 mg/kg or GCM 10 mg/kg. However, after two weeks of treatment, the statistical analysis showed a statistically significant reduction in plasma glucose levels in normoglycemic animals from GCM 3 mg/kg, EAMI 125 mg/kg, EAMI 500 mg/kg, and EAMI 1000 mg/kg groups at times (F (20, 96) = 21.99, p≤0.01 for both) and EAMI 250 mg/kg group only at times of 4 and 6 h (F (20, 96) = 21.99, p<0.0001) when compared to the control group (Fig 4A).

Regarding animals from the diabetic group, the analysis revealed that after 2 weeks of treatment, there was a significant reduction in plasma glucose levels of GCM 10 mg/kg and EAMI 500 mg/kg groups after 4, 6 and 8 h of treatment (F (20, 96% = 28.92, p <0.01 for both) compared to the control group. The decreasing profile persisted for the EAMI 125 mg/kg group after 8 h of treatment (F (20, 96) = 28.92, p <0.01), for the EAMI 250 mg/kg group after 4 and 8 h (F (20, 96) = 28.92, p≤0.01) and for the EAMI 1000 mg/kg group at all times tested (F (20, 96) = 28.92, p <0.05) when compared to the control group. In addition, the same analysis demonstrated a significant increase in blood glucose after 4, 6, and 8 h for the EAMI 125 mg/kg group (F (20, 96) = 28.92, p <0.01) and after 6 and 8 h for the EAMI 250 mg/kg group (F (20, 96) = 28.92, p <0.01) when compared to the GCM 10 mg/kg group (Fig 4B).

**Fig 5:**
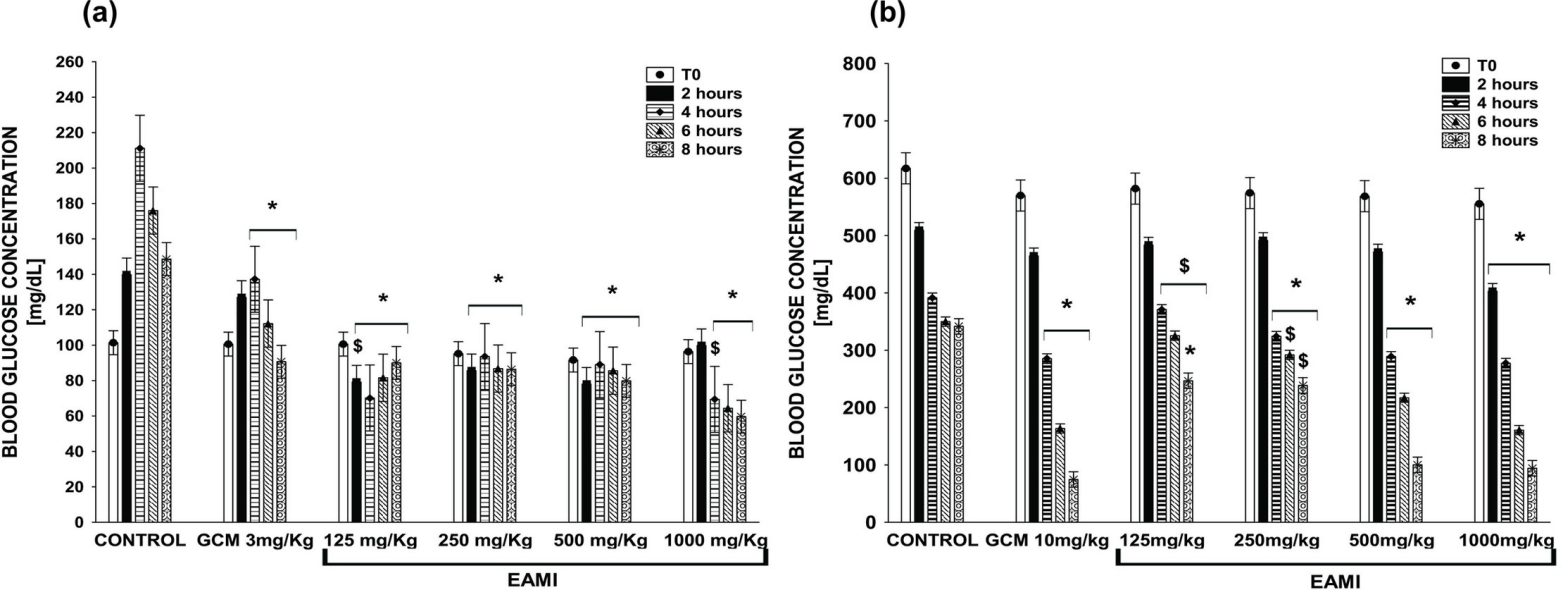
Plasma glucose level after four weeks of treatment. (a) Normoglycemic group; (b) Diabetic group. Bars express the mean ± SEM of the plasma glucose level. Symbols represent significant differences: * differs from the control group; $ differs from the GCM 3 mg/kg or GCM 10 mg/kg group (ANOVA for multivariate measures, p <0.05, *post hoc* Tukey).

After four weeks of EAMI treatment, plasma glucose levels significantly decreased after 2, 4, 6, and 8 hours of treatment for normoglycemic animals from the EAMI 125 mg/kg, EAMI 250 mg/kg and EAMI 500 mg/kg groups (F (20, 96) = 18.32, p≤0.01 for both), when compared to the control groupFor GCM 3 mg/kg and EAMI 1000 mg/kg groups, blood glucose reduction occurred after 4, 6, and 8 hours of treatment (F (20, 96) = 18.32, p <0.05 and (F (20, 96) = 18.32, p <0.01), respectively). In addition, for EAMI 125 mg/kg and EAMI 1000 mg/kg groups after 2 and 4 hours of treatment, respectively, there was a significant reduction of blood glucose when compared to the GCM 3 mg/kg group (F (20, 96) = 18.32, p <0.01, for both), suggesting that the extract exceeded glibenclamide in reducing blood glucose rate (Fig 5A). As for the diabetic group after four weeks of treatment, a decreasing in plasma glucose levels was observed after 4, 6, and 8 hours of treatment for the GCM 3 mg/kg, EAMI 250 mg/kg and EAMI 500 mg/kg groups F (20, 96) = 18.32, p <0.01 for both), after 8 hours for the EAMI 125 mg/kg group (F (20, 96) = 18.32, p <0.01) and at all times tested for the EAMI 500 mg/kg group (F (20, 96) = 18.32, p≤0.01) when compared to the control group. In addition, the same analysis revealed a significant increasing in blood glucose after 4, 6, and 8 hours of treatment for the EAMI 125 mg/kg group (F (20, 96) = 18.32, p <0.01) and after 6 and 8 hours for the EAMI 250 mg/kg group (F (20, 96) = 18.32, p <0.01) when compared to the GCM 10 mg/kg group (Fig 5B).

**Fig 6:**
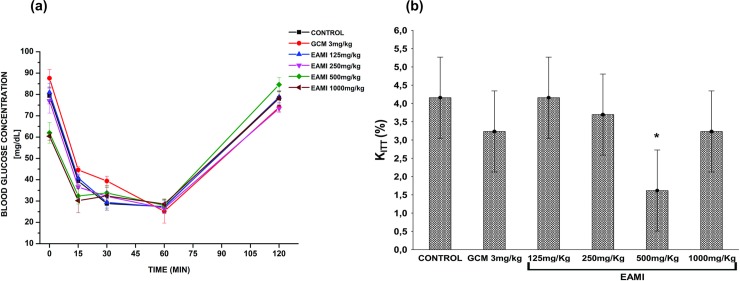
Insulin tolerance test in the normoglycemic group after four weeks of treatment. (a) Plasma glucose level; (b) Percent glucose decay constant (KITT%). Bars express the mean ± SEM of the plasma glucose level. Symbols represent significant differences: * differs from the control group (one-way ANOVA, p <0.05, *post hoc* Tukey).

The ITT has been performed to determine the level of insulin tolerance after four weeks of treatment with EAMI. There was no significant difference in plasma glucose levels for EAMI treated groups and glibenclamide at all times tested when compared to the control group (F (20, 96) = 2.946, p >0.05, for all tested groups) (Fig 6A). Besides, the analysis of the KITT% showed no significant difference between the groups treated with EAMI and glibenclamide and the control group (F (5, 24) = 3.083, p >0.05, for all tested groups), except for the EAMI 500 mg/kg group that exhibited a significant decreasing when compared to the control group (F (5, 24) = 3.083, p = 0.02) (Fig 6B), suggesting that for this group, insulin sensitivity was reduced.

**Fig 7:**
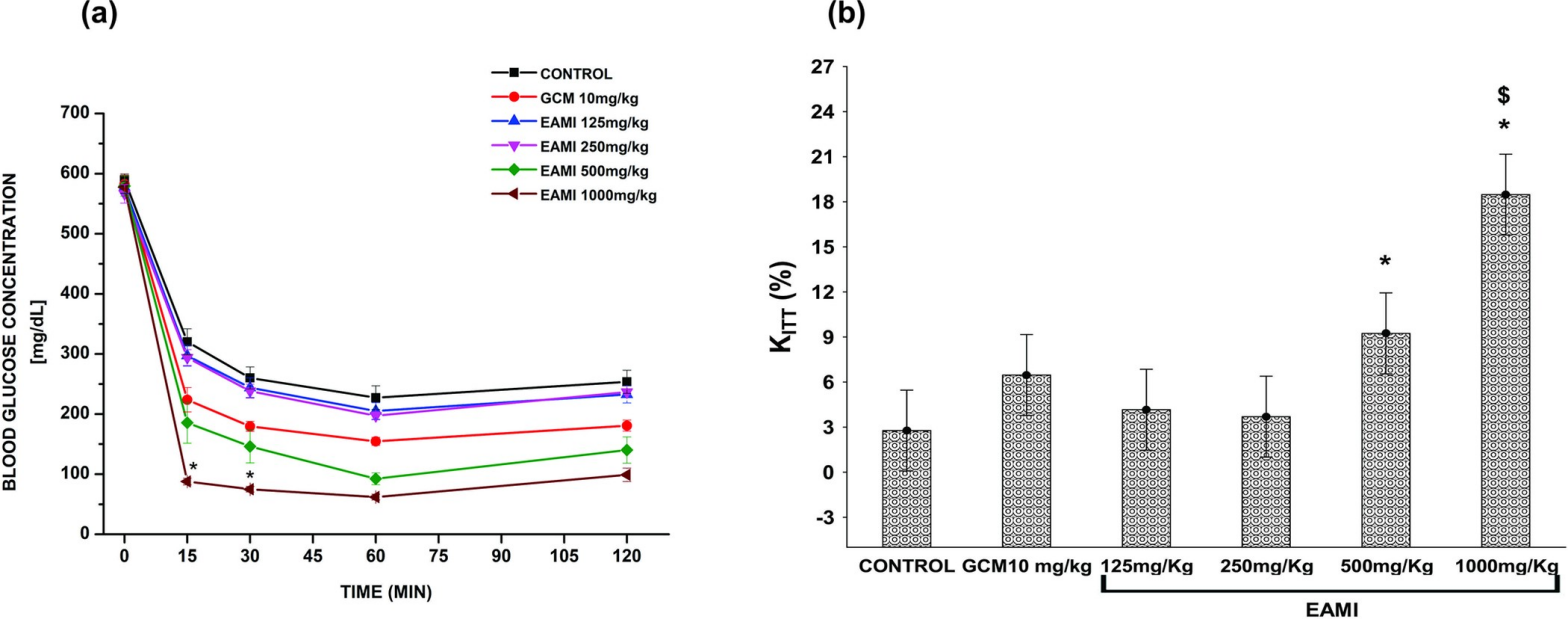
Insulin tolerance test in the diabetic group after four weeks of treatment. (a) Plasma glucose level; (b) Percent glucose decay constant (KITT%). Bars express the mean ± SEM of the plasma glucose level. Symbols represent significant differences: * differs from the control group; $ differs from the GCM 10 mg/kg group (one-way ANOVA, p <0.05, *post hoc* Tukey).

Regarding the ITT analysis for the diabetic group, there was a reduction in plasma glucose levels for the group EAMI 1000 mg/kg after 15 (F (20, 96) = 16.185, p <0.01) and 30 (F (20, 96) = 16.185, p <0.03) minutes after injection of regular insulin, when compared to the control group (Fig 7A). The same analysis revealed a significant increase in KITT% for the EAMI 500 mg/kg and 1000mg/kg groups when compared to the control group (F (5, 24) = 20.291, p = 0.01 and F (5, 24) = 20.291, p <0.01, respectively). Besides, KITT% of the EAMI 1000 mg/kg group was significantly increased when compared to the GCM 10 mg/kg group and the other groups treated with EAMI (F (5, 24) = 20.291, p <0.01, for all groups) (Fig 7B). These results suggest that treatment with EAMI, at the highest doses tested (500 and 1000 mg/kg), significantly increased the sensitivity of animals to insulin, since the higher the KITT% value, the greater the insulin sensitivity.

**Fig 8:**
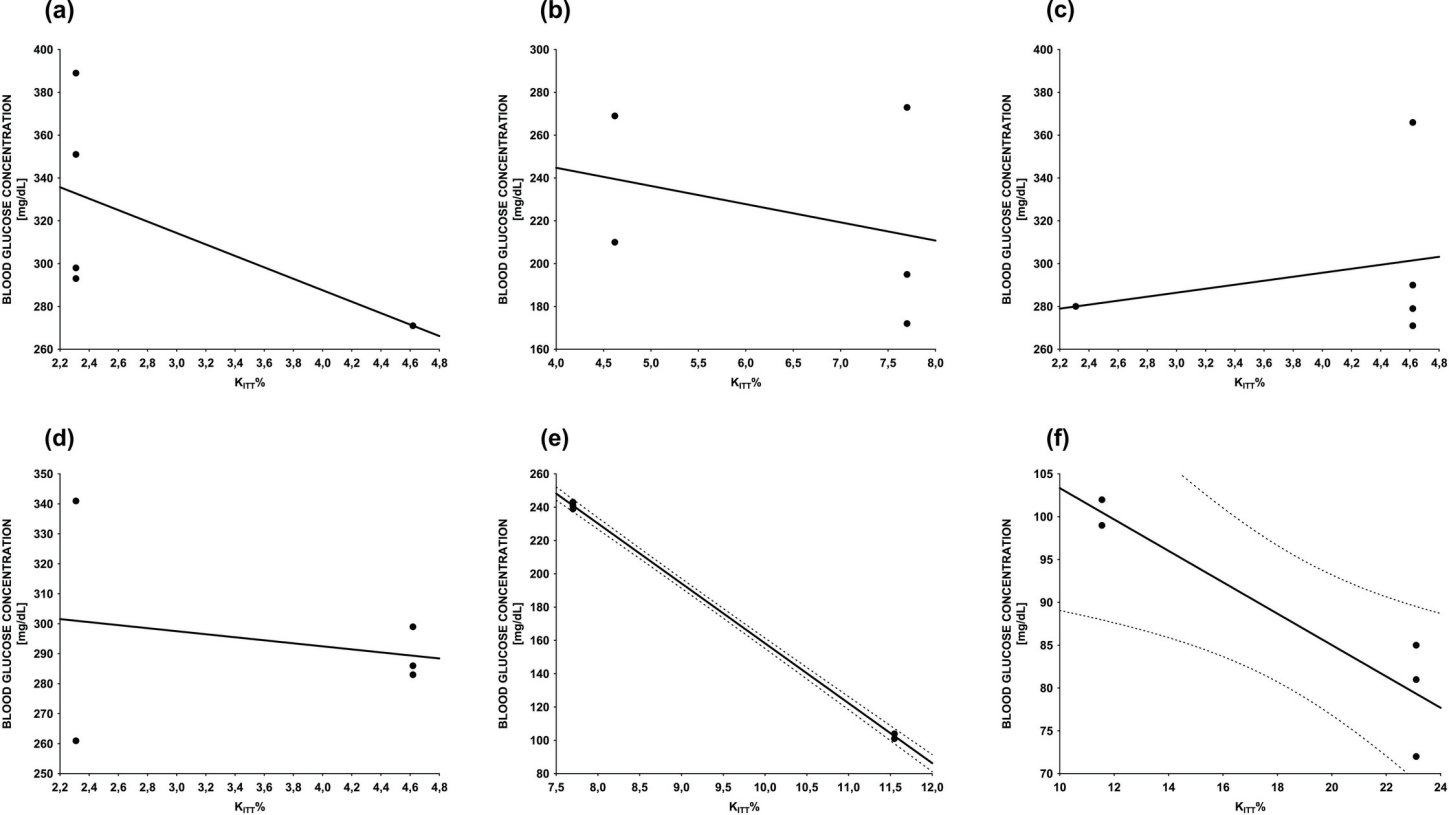
Correlation between percent glucose decay constant (KITT%) and plasma glucose levels during Insulin Tolerance Test (ITT) for diabetics animals. (a) Control group; (b) GCM 10mg/kg group; (c) EAMI 125mg/kg group; (d) EAMI 250mg/kg group; (e) EAMI 500mg/kg group; (f) EAMI 1000mg/kg group (Sperman's correlation test, p <0.05).

Statistical analysis showed a strong negative correlation between KITT% and plasma glucose levels for diabetic animals treated with EAMI at doses of 500 (r = -1.0, p <0.05) and 1000 mg/kg (r = -0.92, p <0.05) during ITT, ie, as KITT% increases, glucose levels decrease (Fig 8E and 8F). This correlation was not significant for the other treatments (r _CONTROL_ = -0.57, p > 0.05; r _GCM 10mg/kg_ = -0.32, p > 0.05; r _EAMI 125mg/kg_ = 0.25, p > 0.05; r _EAMI 250mg/kg_ = -0.22, p > 0.05) (Fig 8A, 8B, 8C and 8D).

**Fig 9:**
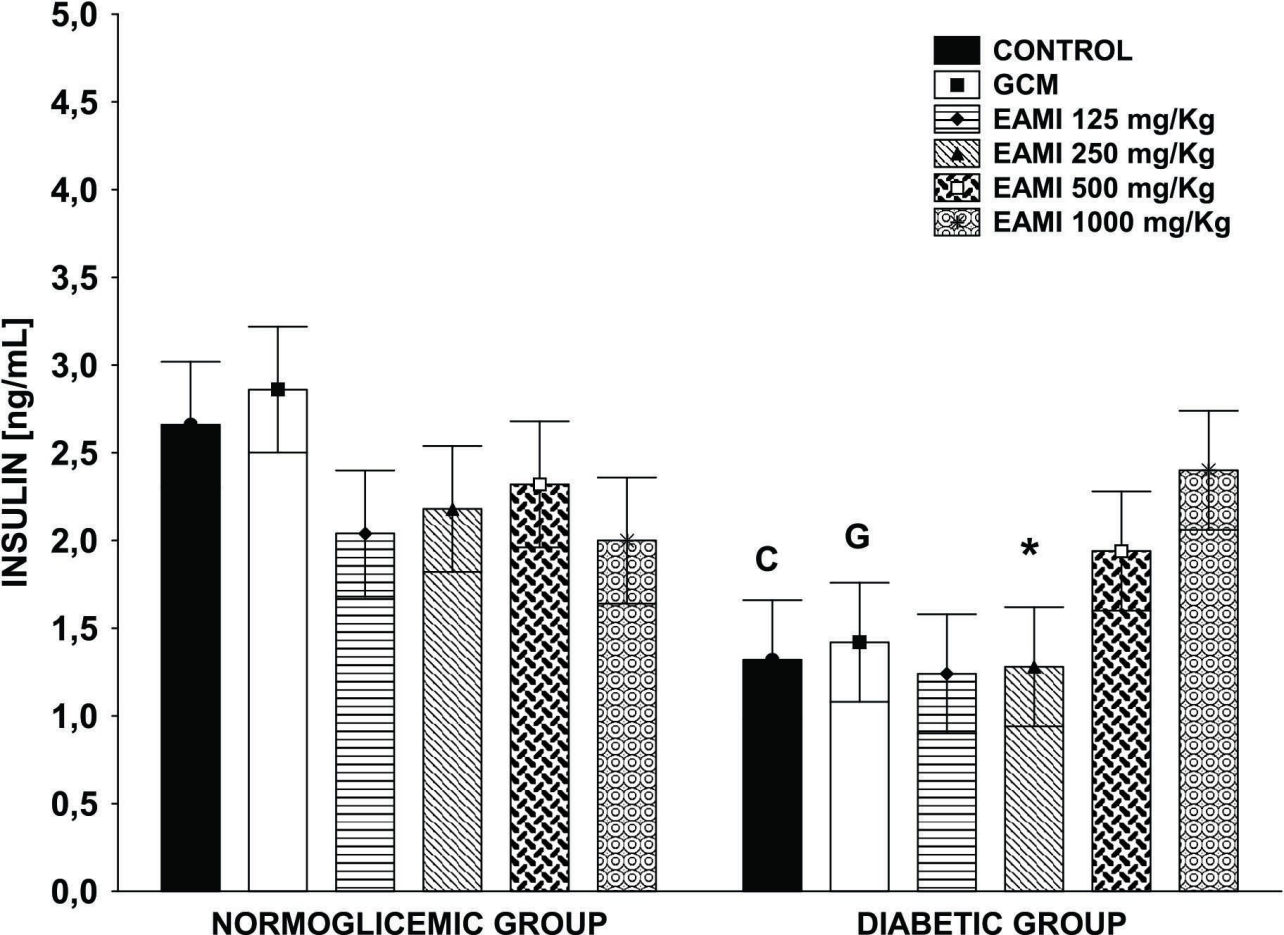
Plasma insulin level after four weeks of treatment. Bars express the mean ± SEM of the plasma glucose level. Symbols represent significant differences: C differs from the control group of normoglycemic animals; G differs from the GCM group of normoglycemic animals; * differs from the EAMI 250 mg/kg group of normoglycemic animals (ANOVA for multivariate measures, p <0.05, *post hoc* Tukey).

Regarding plasma insulin level, the normoglycemic group presented no difference was found between groups treated with EAMI and glibenclamide compared to the control group (F (5, 24) = 8.133, p >0.05, for all tested groups). However, for the diabetic animals, plasma insulin level has been reduced in the control group, glibenclamide, and EAMI 250 mg/kg when compared to the same groups of normoglycemic animals (F_CONTROL_ (5, 24) = 8.133, p <0.01; F_GCM 10mg/kg_ (5, 24) = 8.133, p <0.01; F_EAMI 250mg/kg_ (5, 24) = 8.133, p = 0.03) (Fig 9). Also, the results for diabetic animals treated with EAMI 500 mg/kg and 1000 mg/kg (F_EAMI 500mg/kg_ (5, 24) = 8.133, p = 0.89 and F_EAMI 1000mg/kg_ (5, 24) = 8.133, p = 0.86, respectively) did not demonstrate significant changes in plasma insulin level when compared to the normoglycemic animals, suggesting that the extract has been more effective in reestablishing insulinemia in this group than glibenclamide (Fig 9).

## 4. Discussion

Experimental diabetes models are currently well established. Diabetes induced by STZ, a nitrosourea derivative isolated from *Streptomyces achomogenes*, is trigged by interferences in glucose transport and breakages in cell DNA. A single STZ dose is capable of producing diabetes in rodents due to direct toxic effects on pancreatic endocrine cells. [49]. In the present study, diabetes induction protocol with dose of 100 mg/kg/day for three consecutive days was successful in inducing hyperglycemia.

Plants and their bioactive constituents are used as DM therapy in countries where there is no access to conventional treatment. Several studies report the hypoglycemic activity of *M*. *indica* in animal models of DM [25,26,29,50], although, in most cases, only the acute effect of extracts is demonstrated [23,34,35].

Contrary to these findings, in the present study, no acute effect of treatment with EAMI was observed, since there was no reduction in blood glucose levels. On the other hand, after two and four weeks of daily therapy with EAMI, there was an important hypoglycemic effect, surpassing glibenclamide in reducing the blood glucose rate.

From these data, it is possible to observe face validity (due to the similarity between phenotype (hyperglycemia), predictive validity (due to attenuation of hyperglycemia from clinically effective antidiabetic treatment, glibenclamide), etiological validity (due to triggering of DM by events that are known to induce it in humans, such as the destruction of β-pancreatic cells) and the construct validity (since the pathophysiological bases are similar) of the model used in our laboratory, as well as the potential hypoglycemic effect of EAMI.

Long-term treatments prolong life, alleviate symptoms, and reduce the risks inherent to DM [9]. Our group is one of the pioneers to demonstrate the long-term maintenance of the hypoglycemic effect of EAMI. The results of the present study showed that EAMI was effective in maintaining the hypoglycemic effect over four weeks of treatment.

The reduction in plasma glucose levels, increased insulin sensitivity, and increased plasma insulin concentration observed in the study can be attributed to the phenolic compounds present in EAMI, especially flavonoids. Previous studies have shown that the antidiabetic effect of extracts obtained from different *M*. *indica* parts can be partially attributed to its ability to inhibit α-amylase and α-glycosidase, enzymes that hydrolyze carbohydrates in the intestine. Possibly, due to the presence of tannins and phenolic compounds that inhibit α-amylase and α-glucosidase activities [51].

Here, high levels of phenolic compounds, tannins and five flavonoids were isolated from the EAMI (**F-1** Kaempferol; **F-2** Fisetin; **F-3** Galangin; **F-4** Chrysin and **F-5** Luteolin).

A recent study on the mechanism of action of the ethanolic extract obtained from *M*. *indica* leaves has shown its dose-dependent inhibitory ability on alpha-amylase enzyme activity. Alpha-amylase plays a crucial role in the hydrolysis of alpha-1,4 glycosidic linkages of carbohydrates which triggers to the breakdown of large starch molecules in oligosaccharides to cross the intestinal epithelium. Besides, the same study demonstrated that the extract: **1)** is capable of adsorbing glucose molecules in the gut preventing their systemic absorption. In this case, the adsorption capacity of the extract was attributed to its phytoconstituents as insoluble and soluble fibers capable of adsorbing glucose; **2)** increases glucose uptake in LO-2 cells when compared to control [29]; however, this glucose uptake capacity was lower than that of metformin and *Brachylaena elliptica* [52]. It is well established in the current literature that phytoconstituents such as phenols, terpenoid flavonoids, and flavanols reduce the release and increase glucose uptake, contributing to improved hyperglycemia in type 2 diabetes [53]; **3)** significantly eliminates DPPH and ABTS^+^ radicals at a concentration of 200 μg/mL; however, when compared to vitamin C (positive control), minor free radical scavenging activities [29]. Previous studies have shown that phytochemicals such as vitamins, carotenoids, flavonoids, anthocyanins, tannins, and other phenolic compounds are capable of suppressing DM [54]; **4)** reduced levels of nitric oxide production at different doses. Thus, the ability of the extract to eliminate free radicals and inhibit their production may contribute partially to the prevention of complications in type 2 diabetes [29].

Regarding the biological activities of the flavonoids isolated in the present study, Kaempferol showed good α-glucosidase inhibitory activity in yeast models [55]. Medicinal plants have been used for screening the anti-diabetic agents through varieties models *in vitro*, including inhibition of α-glucosidase and α-amylase [56,57]. Besides, previous studies have shown that fisetin broad pharmacological properties such as anticancer [58], inhibition of angiogenesis [59], antiallergic [60], and antithyroid effects [61]. Fisetin has been reported to downregulate both glycogenolysis and gluconeogenesis *in vitro* [62]. Also, fisetin presents antidiabetic potential in streptozotocin-induced experimental diabetic rats [63,64]. Current studies have shown that administration of galangin reduced hyperlipidemia related to the risk of diabetic complications and could be beneficial for diabetic hyperlipidaemic patients [65]. Besides, galangin decreased oxidative stress and increased antioxidant status in diabetic rats, which may be due to its antidiabetic and antioxidant potential [66]. Data from the literature have shown that chrysin could produce similar effects as metformin, a drug used for the treatment of diabetes [67] and that administration of luteolin was effective on improvement of diabetes in KK-Ay mice [68]. Thus, the flavonoids isolated from EAMI, conclusively, are involved with the effects found in the present research.

The effects observed at the different EAMI doses were similar to those of glibenclamide at practically all times analyzed, in both normoglycemic and diabetic animals. Glibenclamide is a second-generation sulfonylurea that stimulates the insulin-releasing by pancreatic β-cell due to the inhibition of ATP-sensitive potassium channels [69]. These data corroborate the effectiveness of EAMI during the antidiabetic therapy since they demonstrated similar outcomes to a hypoglycemic used clinically and evidenced the maintenance of the long-term effect.

Insulin resistance is a metabolic change inherent to type 2 diabetes [70,71], type 1 uncontrolled diabetes [72], diabetic ketoacidosis [73], and obesity [74,75]. The insulin tolerance test evaluates the rate of glucose decay over 120 minutes after insulin injection. The decrease in glucose levels is determined by two factors: 1) Suppression of hepatic glucose production and; 2) Due to the stimulation of glucose uptake by insulin-sensitive tissues. The interpretation of K_ITT_% is based on the speed and intensity of glucose reduction, and the faster and more intense, the more sensitive the individual is to the action of insulin [76].

Disagreements regarding the KITT% model refer to the possibility of activation of a counter-regulatory response by hypoglycemia on hormones that could influence on glucose depletion, such as glucagon, catecholamines, and growth hormone. However, this counter-regulatory response usually appears only 15 to 20 minutes after insulin injection [77,78]. Thus, the decrease in glucose observed within the first 15 minutes after the start of the test reflects insulin-induced uptake of glucose by tissues, as well as inhibition of glucose release by the liver [78]. In the present study, that effect was observed in diabetic animals treated with EAMI 1000 mg/kg within the first 15 minutes after insulin injection, suggesting that the extract induced increased glucose uptake by insulin-sensitive tissues and suppression of hepatic glucose production. The correlation between plasma glucose levels during ITT and KITT% corroborates the hypothesis of a dose-dependent increase in insulin sensitivity in animals treated with EAMI (500 and 1000 mg/kg), as increased KITT% promotes reduced glucose levels.Fasting insulin has been identified as a simple method for assessing insulin sensitivity throughout the body, especially in large populations. In clinical practice, insulin dosage in diabetics, if reduced, may not indicate low insulin resistance but rather a failure in pancreatic beta-cells function [76]. In our study, we demonstrated that insulin is significantly increased in EAMI treated animals (500 and 1000 mg/kg) animals, suggesting that the possible action mechanism exerted by the compounds present in the extract is the increase of insulin secretion. Flavonoids and other phenolic compounds may act in this way, as described above.

## 5. Conclusion

The present study demonstrated that EAMI has a high content of total phenolic compounds, total tannins, and, mainly, flavonoids. After oral administration, the extract induced a hypoglycemic effect in diabetic rats after both two and four weeks of treatment. The extract was useful in maintaining the long-term effect, characterizing an antidiabetic therapy.The EAMI significantly increased dose-dependent insulin sensitivity in diabetic animals, as well as the plasma insulin level. Despite the flavonoids isolated from EAMI, conclusively, are involved with the effects found in the present research, future research should be aimed at examining the impact of these compounds in isolation and confirm the hypotheses raised. Hence, it will allow the development of new antidiabetic therapies with lower adverse effects, maximized access to the population, and greater adherence of patients to treatment.

## Abbreviations

CE: catechin equivalent
DM: Diabetes mellitus
EAMI: aqueous extract from M. indica
GAE: gallic acid equivalent
GCM: Glibenclamide
ITT: Insulin Tolerance Test
KITT%: Percent glucose decay constant
LETOX: Laboratory of Toxicological Trials
NC: negative control
PEG 6000: polyethylene glycol 6000
RIA: Radioimmunoassay
SEM: standard error of the mean
STZ: streptozotocin
TLC: thin-layer chromatography
UFGD: Federal University of Grande Dourados
UFMS: Federal University of Mato Grosso do Sul
UNIGRAN: University Center of Grande Dourados
